# Key Aspects of Myo-Inositol Hexaphosphate (Phytate) and Pathological Calcifications

**DOI:** 10.3390/molecules24244434

**Published:** 2019-12-04

**Authors:** Felix Grases, Antonia Costa-Bauza

**Affiliations:** Laboratory of Renal Lithiasis Research, University Institute of Health Sciences Research (IUNICS-IdISBa), University of Balearic Islands, Ctra Valldemossa, km 7.5, 07122 Palma de Mallorca, Spain; antonia.costa@uib.es

**Keywords:** myo-inositol hexaphosphate, inositol phosphates, calcium renal calculi, cardiovascular calcification, tissue calcification, osteoporosis

## Abstract

Phytate (myo-inositol hexaphosphate, InsP6) is an important component of seeds, legumes, nuts, and whole cereals. Although this molecule was discovered in 1855, its biological effects as an antinutrient was first described in 1940. The antinutrient effect of phytate results because it can decrease the bioavailability of important minerals under certain circumstances. However, during the past 30 years, researchers have identified many important health benefits of phytate. Thus, 150 years have elapsed since the discovery of phytate to the first descriptions of its beneficial effects. This long delay may be due to the difficulty in determining phytate in biological media, and because phytate dephosphorylation generates many derivatives (InsPs) that also have important biological functions. This paper describes the role of InsP6 in blocking the development of pathological calcifications. Thus, in vitro studies have shown that InsP6 and its hydrolysates (InsPs), as well as pyrophosphate, bisphosphonates, and other polyphosphates, have high capacity to inhibit calcium salt crystallization. Oral or topical administration of phytate in vivo significantly decreases the development of pathological calcifications, although the details of the underlying mechanism are uncertain. Moreover, oral or topical administration of InsP6 also leads to increased urinary excretion of mixtures of different InsPs; in the absence of InsP6 administration, only InsP2 occurs at detectable levels in urine.

## 1. Introduction

Phytate (myo-inositol hexaphosphate, InsP6) is an important dietary component of many edible seeds, legumes, nuts, and whole cereals and generally occurs as a calcium/magnesium salt. The major food sources of phytate typically contain 0.5% to 3% of dry weight as phytate [1,2,3,4]. Other inositol phosphates, such as inositol pentaphosphates (InsP5s) and inositol tetraphosphates (InsP4s), occur at lower levels in these plant foods (<15% of all InsPs) [5]. Diets rich in legumes, nuts, and whole grains provide an important source of phytate. Thus, the Mediterranean diet provides 1 g to 1.5 g of daily phytate as a calcium/magnesium salt (also known as phytin), much more than diets with refined cereals. The European/American diet can supply a broad range of 0.2 g to 1.5 g of daily phytate, depending on consumption of legumes, nuts, and whole cereals [6]. Administration of high phytate doses must be adequately controlled and the content of minerals must be considered. When balanced diets contain adequate amounts of legumes, whole cereals and nuts, as in the Mediterranean diet, the phytate supplied by these foods is enough to maintain adequate levels in the organism and no negative effects on the mineral balance is produced [6].

Phytate was discovered in 1855–1856 [7,8] and its structure was determined in 1914 [9]. In the 1940s, the first physiological experiments described phytate as an antinutrient, because it reduced the absorption of trace elements, such as Zn and Fe (III), with which it can form insoluble compounds [10,11,12]. This effect occurs primarily when phytate is supplied in large amounts with unbalanced diets, in which case the formation of insoluble compounds reduces the absorption of trace elements. However, consumption of phytate in moderate amounts with balanced diets does not reduce the bioavailability of these essential elements [13,14]. During the last 30 years, many studies have described important positive effects of phytate on health. In particular, phytate can function as an antioxidant [15], has anticancer activity [16,17], prevents renal lithiasis and pathological calcifications [18], reduces the glycemic index [19], and normalizes the levels of glucose and cholesterol [20,21].

**Figure 1:**
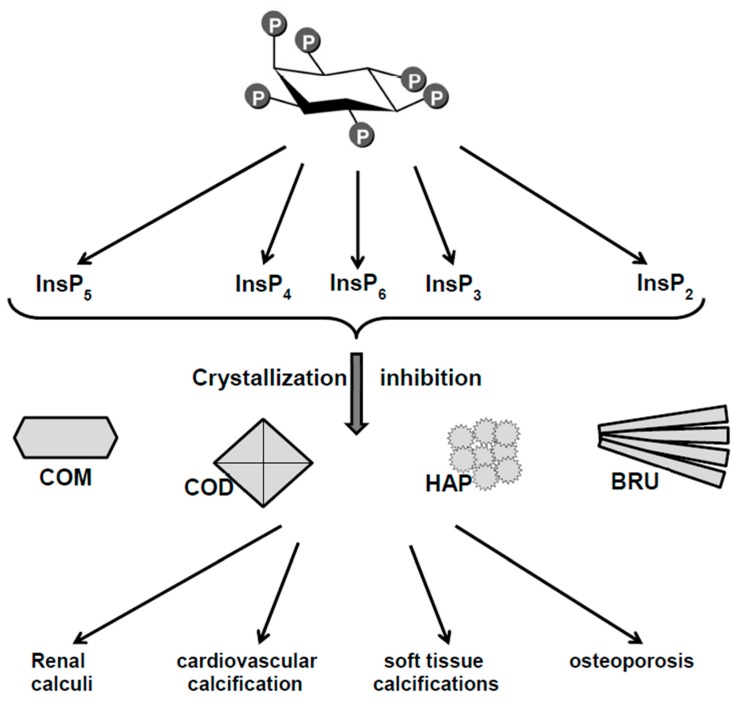
Phytate as crystallization inhibitor of biological calcium salts. COM: Calcium oxalate monohydrate, COD: calcium oxalate dihydrate, HAP: Hydroxyapatite, BRU: Brushite.

Considering the many clinical benefits of phytate, it may seem surprising that about 150 years elapsed from the discovery of the molecule until the identification of these benefits. Some of the reasons for this long delay may be that phytate has a low molecular weight, occurs at low concentrations in biological media, is difficult to identify and quantify, and dephosphorylation can generate a significant number of derivatives (InsPs) which also have important biological functions (Figure 1). This review will analyze the role of phytate and other InsPs as inhibitors of the crystallization of calcium salts, and the relationship between phytate consumption with the development of pathological calcifications and excretion of InsPs.

## 2. Phytate (InsP6) and Inositol Phosphates (InsPs) as Crystallization Inhibitors of Calcium Salts: In Vitro Studies

Partially hydrolyzed phytic acid is a potent inhibitor of hydroxyapatite formation in vitro [22]. Partial hydrolysates of phytate, which contain mixtures of different InsPs, are potent inhibitors of in vitro calcification of rat cartilage, and parenteral injection prevents aortic calcification in rats treated with high-dose vitamin D [23]. Diverse studies have shown that phytate inhibits the crystallization of calcium into oxalate and phosphate salts. Some of these studies were performed under conditions that mimic the formation of kidney stones or pathological tissue calcifications. For example, a study of early-stage renal stone formation examined the urothelium of the pig urinary bladder under diverse conditions, such as eliminating the protective layer of glycosaminoglycans and using free radicals to generate urothelial lesions. In all cases, phytate inhibited the development of calcifications [24,25]). Other in vitro studies that simulated the early stages of renal stone formation using flow systems also demonstrated that phytate prevented the development of calcium oxalate crystals [26]. A study of inhibitors of the heterogeneous nucleation of calcium oxalate monohydrate (COM) on different solid substrates (calcium phosphate, mixture of mucin and calcium phosphate, wax), demonstrated that only phytate totally inhibited COM formation [27]. A study of the interactions of phytate with other well-known stone inhibitors, such as magnesium and citrate, reported synergistic effects only between magnesium and phytate [28]. Phytate also efficiently inhibited stone growth in the presence of macromolecules in vitro, consistent with the presence of urinary excretion of phytate in vivo [29]. Finally, a thermodynamic and kinetic analysis of phytate as crystallization inhibitor found it altered the kinetics but not the thermodynamics of stone formation [30].

These many studies clearly indicate that InsP6 and InsPs, as well as pyrophosphate and other polyphosphates as bisphosphonates, can effectively inhibit calcium salt crystallization. This inhibitory effect must be attributed to the high affinity of its phosphate groups for calcium in crystalline growth-active sites. It is interesting to consider that bisphosphonates were first synthesized in the 19th century, but their biological effects were not discovered until the 1960s and were related to the inhibition of vascular calcification [31,32]. At present, as it is well known, they are used primarily to prevent bone resorption in the treatment of osteoporosis [33].

## 3. Relationship Between Oral or Topical Intake of Phytate and Pathological Calcifications

### 3.1. Animals

A study using male Wistar rats examined the development of papillary calcifications induced by addition of ethylene glycol to drinking water. The supplementation of this water with sodium phytate significantly reduced the development of calcifications at the tips of the papillae and the total calcium content of the papillary tissue [34].

AIN-76A is a purified rodent diet which contains no phytate, and promotes the development of renal calcifications in female rats. Researchers compared a group of animals fed this diet with another group that received AIN-76A + 1% InsP6 (the same percentage of InsP6 in the common rodent diet). They found that mineral deposits at the corticomedullary junction only occurred in animals that received AIN-76A alone [35].

Other study used nicotine and vitamin D to induce calcification in the renal and cardiovascular tissues of male Wistar rats that were fed AIN-76A diet, a purified diet in which phytate is undetectable. Untreated rats developed significant calcium deposits in the kidneys, aorta, and heart, but rats treated with a moisturizing cream containing 2.0% potassium salt of InsP6 had no deposits or significantly decreased deposits [36,37]. Calcium deposits were examined by histological analysis (after hematoxylin and eosin staining) and by calcium quantification through chemical analysis by ICP-AES. Moisturizing cream was topically administered. The application skin surface on the back was shaved every four days. Phytate was readily absorbed from skin but the exact mechanism of absorption is unknown. Significant increase of urinary InsPs was detected in animals treated with the moisturizing phytate cream.

To evaluate the effects of dietary phytate on cardiovascular calcification in rats during aging, researchers fed one group of male Wistar rats with a balanced diet (UAR-A04) containing InsP6, fed a second group with AIN-76A that was enriched with the calcium–magnesium salt of InsP6, and fed a third group with AIN-76A alone. Sacrifice of the animals at the age of 1.5 years indicated that the levels of calcium in the aorta were significantly lower in the two groups that received InsP6, thus demonstrating that long-term dietary intake of InsP6 significantly reduced age-related aorta calcification [38].

Other experiment studied soft tissue calcification in rats that received calcinosis induction by subcutaneous injection of a potent oxidant (KMnO_4_). One group of animals received the AIN-76A diet and the other group received the same diet with a calcium–magnesium salt of InsP6. Calcification was significantly reduced in rats that received the diet with InsP6 [39,40]. A similar study that used a moisturizing cream with a 2% InsP6 as a sodium salt (rather than a dietary supplement) reported significantly reduced calcifications compared with the control group [41].

Bone decalcification, which occurs during osteoporosis, may be considered the opposite of calcification. However, some agents that prevent clinical decalcification, such as bisphosphonates, also inhibit calcification. This is in part because the powerful interaction of calcium and phosphate hinders crystallization and crystal redissolution. Thus, a study examined the influence of consumption of the Ca–Mg salt of InsP6 on the characteristics of bones in ovariectomized rats (an animal model for postmenopausal osteoporosis). These researchers fed one group with AIN-76A and another group with AIN-76 enriched with 1% Ca–Mg salt of InsP6. After 12 weeks the calcium and phosphorous content and bone mineral density were significantly greater in the femoral bones and L4 vertebrae of rats that received InsP6. Because Ca–Mg–InsP6 consumption reduces bone mineral density loss due to estrogen deficiency, InsP6 thus exhibits effects similar to those of bisphosphonates on bone resorption [42]. In fact, InsP6 and bisphosphonates each inhibit osteoclastogenesis and mineralization of osteoblasts [43].

The results of these many studies indicated that oral or topical administration of InsP6 to experimental animals under different conditions significantly decreased the development of pathological calcifications. Although these studies have not examined the mechanism of this effect, they nonetheless demonstrated that administration of InsP6 decreased pathological calcification in vivo.

### 3.2. Humans

A prospective study examined the association between dietary factors and the risk of incident kidney stones in 96,245 females. The results indicated that ingestion of dietary InsP6 significantly reduced the risk of calcium stones, and the researchers concluded that InsP6 should be considered an important and safe addition to the diet for prevention of stone formation [44].

Another study examined three groups of calcium oxalate stone-formers: One group received no treatment, the second group received Ca–Mg–InsP6, and the third group received potassium citrate. The researchers measured urinary lithogenic risk before and after treatment (15 days) using a test specially designed for this purpose. A significant number of oxalocalcic stone-formers had urine with high lithogenic risk at the beginning. After treatment, 52% of patients in the citrate group and 50% of patients in the Ca–Mg–InsP6 group had decreased risk, but only 7% of untreated individuals had a decreased risk [45].

A prospective cross-sectional study of abdominal aortic calcifications in patients with chronic kidney disease found that patients with no or only mild abdominal aortic calcifications had lower pulse pressure, greater intake of InsP6, greater urinary InsPs, and a lower prevalence of prior cardiovascular disease than those with moderate or severe abdominal aortic calcification [46].

A study of 433 subjects measured bone mineral density in the lumbar column and the neck of the femur, and individually interviewed subjects about selected osteoporosis risk factors. Dietary information on InsP6 consumption was obtained by questionnaires conducted under two different occasions. The results indicated that bone mineral density increased with the greatest InsP6 consumption. Multivariate linear regression analysis indicated that body weight and low InsP6 consumption were the risk factors with greatest influence on bone mineral density. These results thus suggest that InsP6 consumption can protect against the development of osteoporosis [47].

The results of these human studies are consistent with those of the animal studies, in that the oral ingestion of InsP6 provides protection against pathological calcification–decalcification processes.

## 4. Relationship Between Intake of InsP6 and Excretion of InsPs

The difficulty of identifying and quantifying InsP6 and its dephosphorylated products in biological media is one reason it has taken so long to identify its beneficial effects. The most common analytical methods for quantifying InsP6 are based on non-specific measurements of total inorganic phosphate or complex formation by phosphate groups [48,49,50,51]. In some cases, these procedures require prior separation processes. Because these methods are non-specific, they can overestimate the amount of InsP6 when other InsPs are present in a sample [52]. These methods are nevertheless very useful when a non-specific global estimation of InsPs is sufficient.

Currently, the coupling of high performance liquid chromatography (HPLC) with mass spectrometry (MS) in its most current variants seems to provide better results [53,54]. One of the limitations of this method is the lack of available standards for many of the InsP isomers.

Below, we discuss the relationship between administration of oral or topical InsP6 and excretion of InsPs.

### 4.1. Animals

Researchers studied the relationship between oral InsP6 and InsPs excretion in two groups of male Wistar rats: One group received tap water and normal rat food pellets (InsP6 rich diet); the other group received a liquid diet in which InsP6 was initially absent, but there was a gradual increase of dietary InsP6 over time [55]. When InsP6 was absent from the diet, the urinary excretion of InsPs declined to undetectable levels after 22 days. The addition of increasing amounts of InsP6 to the liquid diet led to increased urinary excretion of InsPs. Although urinary excretion was dependent on the oral dose, ingestion of more than 20.9 mg/kg per day led to no additional excretion.

Another study measured urinary InsPs after topical administration of InsP6 to female Wistar rats [52]. These researchers measured all urinary InsPs using a non-specific method, measured the level of InsP6 using a specific method (polyacrylamide gel electrophoresis), and identified the different urinary InsPs using MS. After dietary deprivation of InsP6, InsP2 was the only detectable InsP in the urine. Rats that received topical InsP6 treatment had abundant InsP6 and other InsPs in their urine (InsP5, InsP4, InsP3, InsP2). Moreover, the amounts of urinary InsP6 was highly variable among animals. The results were similar following oral administration of InsP6, although the urinary levels of the different InsPs were lower [56].

### 4.2. Humans

A study examined healthy volunteers who received an InsP6-depleted diet during an initial period, and an InsP6-normal diet during a subsequent period. The InsP6-depleted diet led to lower plasma levels of InsPs than the InsP6-normal diet. After the onset of the InsP6-normal period, normal plasma and urinary InsPs levels were achieved in 16 days [57].

Another study compared participants who consumed a Mediterranean diet that had a low proportion of InsP6-rich foods with other participants who consumed a Mediterranean diet that had a high proportion of InsP6-rich foods. The overall InsP6 consumption of the first group corresponded to 422 ± 34 mg per day, and that of the second group was 672 ± 50 mg per day, representing a 59% difference. Unsurprisingly, urinary InsPs excretion was significantly higher (54%) in the second group [58].

Thus, studies of animals and humans agree that increased oral or topical administration of InsP6 leads to increased urinary excretion of total InsPs, and to excretion of mixtures of multiple different InsPs.

## 5. Conclusions and Future Trends

The in vivo functions of InsP6 and the products that result from its dephosphorylation (InsP5, InsP4, InsP3, and InsP2) are similar to those of pyrophosphate and bisphosphonates, in that they are potent inhibitors of the formation of calcium oxalate and calcium phosphate crystals (Figure 1).

The effect of InsP6 administration to animals and humans on inhibition of the development of pathological calcifications, is associated with the appearance of mixtures of different InsPs in blood and urine. Thus, in the absence of oral or topical administration of InsP6, only InsP2 is detectable in urine. Thus, we suggest that InsP6 should be considered an “anticalcification” vitamin.

In spite of improvements in the analytical determination of InsP6 and its dephosphorylated derivatives, many aspects of the chemical analysis of InsPs require further study. Due to the large number of isomers produced by dephosphorylation of InsP6 it is obviously necessary to develop specific analytical methods for quantification. On the other hand, it is also important to determine the roles of different enzymes that dephosphorylate InsP6 (phytases and alkaline phosphatases) in the intestinal tract and plasma, because the formation of InsPs with fewer phosphates could favor gastrointestinal absorption. It would be interesting to be able to establish the possible relationship between the levels of alkaline phosphatases in plasma and the profile of urine InsPs. Consequently, it would be important to know if it is possible to modulate InsPs dephosphorilation and which enzyme inhibitors may be involved.

The great variability in the normal levels of alkaline phosphatases that occur in vivo could explain the variability in the distribution of InsPs observed between individuals belonging to the same study [56].
